# Fluorescence-guided surgical system using holographic display: from phantom studies to canine patients

**DOI:** 10.1117/1.JBO.28.9.096003

**Published:** 2023-09-20

**Authors:** Mebin B. George, Benjamin Lew, Zuodong Liang, Steven Blair, Zhongmin Zhu, Nan Cui, Jamie Ludwig, Mohamed Zayed, Laura Selmic, Viktor Gruev

**Affiliations:** a University of Illinois at Urbana-Champaign, Department of Electrical and Computer Engineering, Urbana, Illinois, United States; b University of Illinois at Urbana-Champaign, Division of Animal Resources, Urbana, Illinois, United States; c Washington University in St. Louis, Department of Surgery, St. Louis, Missouri, United States; d Ohio State University, Department of Veterinary Clinical Sciences, Columbus, Ohio, United States; e University of Illinois at Urbana-Champaign, Department of Bioengineering, Urbana, Illinois, United States; f University of Illinois at Urbana-Champaign, Beckman Institute for Advanced Science and Technology, Urbana, Illinois, United States; g University of Illinois at Urbana-Champaign, Carle Illinois College of Medicine, Urbana, Illinois, United States

**Keywords:** fluorescence guided surgery, holographic displays, bioinspired sensors, cancer surgery, goggles

## Abstract

**Significance:**

Holographic display technology is a promising area of research that can lead to significant advancements in cancer surgery. We present the benefits of combining bioinspired multispectral imaging technology with holographic goggles for fluorescence-guided cancer surgery. Through a series of experiments with 43D-printed phantoms, small animal models of cancer, and surgeries on canine patients with head and neck cancer, we showcase the advantages of this holistic approach.

**Aim:**

The aim of our study is to demonstrate the feasibility and potential benefits of utilizing holographic display for fluorescence-guided surgery through a series of experiments involving 3D-printed phantoms and canine patients with head and neck cancer.

**Approach:**

We explore the integration of a bioinspired camera with a mixed reality headset to project fluorescent images as holograms onto a see-through display, and we demonstrate the potential benefits of this technology through benchtop and *in vivo* animal studies.

**Results:**

Our complete imaging and holographic display system showcased improved delineation of fluorescent targets in phantoms compared with the 2D monitor display approach and easy integration into the veterinarian surgical workflow.

**Conclusions:**

Based on our findings, it is evident that our comprehensive approach, which combines a bioinspired multispectral imaging sensor with holographic goggles, holds promise in enhancing the presentation of fluorescent information to surgeons during intraoperative scenarios while minimizing disruptions.

## 1 Introduction

Cancer is a prevalent disease that affects ∼40% of men and women during their lifetime, according to the American Cancer Society.^1^ Although significant progress has been made in improving cancer treatment, approximately one-fifth of patients with cancer still succumb to the disease.^1^ According to the World Health Organization, cancer is the second leading cause of death globally and is responsible for an estimated 10 million deaths each year.^2^ Timely detection and complete resection of all cancerous tissue are crucial factors that determine a patient’s 5-year survival rate. Preoperative imaging instruments, such as MRI, PET/CT, and ultrasound, along with regular screenings, are improving the detection of early stage cancer. However, there is often a disconnect between preoperative surgical information and intraoperative procedures. Surgeons rely on their sight and palpation as primary sensing modalities during surgeries, and their experience is critical to the success of the procedure. Incomplete cancer resection remains a common problem in many types of surgical procedures, as small deposits of cancerous cells in the surrounding tissues and at the surgical margins can blend in with healthy tissue, as evidenced by up to 25% of breast cancer patients, 35% of colon cancer patients, and as many as 40% of head and neck cancer patients experiencing incomplete resection.^3^^–^^6^

Real-time intraoperative imaging techniques have been evaluated through various clinical trials to address these challenges during cancer surgery.^7^^–^^9^ Near-infrared (NIR) fluorescence-guided cancer surgery has demonstrated great potential for improving cancer surgery, with success relying on three essential components: tumor-targeted probes, illumination and imaging devices, and display units.^10^^,^^11^ The advancement of this field from research laboratories to the clinic was initially propelled by the use of passive probes. Indocyanine green (ICG) and methylene blue (MB) fluorophores were among the initial agents employed for angiography, tumor delineation through passive accumulation, and mapping of sentinel lymph nodes (SLNs) in cancer patients.^12^ Since 2021, pafolacianine has been approved globally for imaging ovarian and lung tumors that overexpress folate receptors.^13^^–^^15^ Many other tumor-targeted probes are in phase three clinical trials or have completed them, and commercialization is imminent.^9^^,^^16^^,^^17^

Intraoperative imaging and illumination devices have greatly benefited from advancements in cellphone camera technology and photonics materials.^18^ Miniature imaging sensors have enabled high-resolution, real-time imaging in both visible and NIR spectrums for both open and minimally invasive surgeries.^19^^,^^20^ Single-chip multispectral cameras are providing an alternative to traditional multicamera systems, and meta-surfaces are enabling miniaturization of multispectral sensors for chip-on-a-tip endoscopic applications.^21^^,^^22^ Although laser diode has conventionally been employed as the main source of illumination, there is potential for the integration of novel high-power light emitting diodes (LEDs) for fluorescent-guided surgery (FGS).^23^

Tumor-specific probes, intraoperative imaging, and illumination devices are critical for real-time detection of cancerous tissue in the operating room. However, displaying this information in a time-sensitive manner without interrupting the surgical workflow is equally crucial.^24^ The existing intraoperative imaging instruments approved by the FDA employ conventional monitors, resulting in the loss of depth information. This limitation can potentially contribute to iatrogenic damage, prolonged surgical time, and incomplete tumor resection.

The use of wearable goggles presents a partial solution to address this limitation by allowing surgeons to receive color and NIR fluorescent information in the operating room without altering their line of sight. These goggles work in conjunction with color and NIR imaging sensors, which capture both color images for surgical guidance and NIR fluorescent information to identify tumor locations. The color and NIR images are merged into a single real-time image, which is then presented to the surgeon. It is important to note that these synthetic images are not directly aligned with the surgeon’s natural eyesight but are instead displayed near the surgical site. The efficacy of this technology has been evaluated in intraoperative settings, specifically in patients with breast, melanoma, or liver cancer, using ICG as the NIR fluorescent probe.^25^^–^^30^

**Fig. 1:**
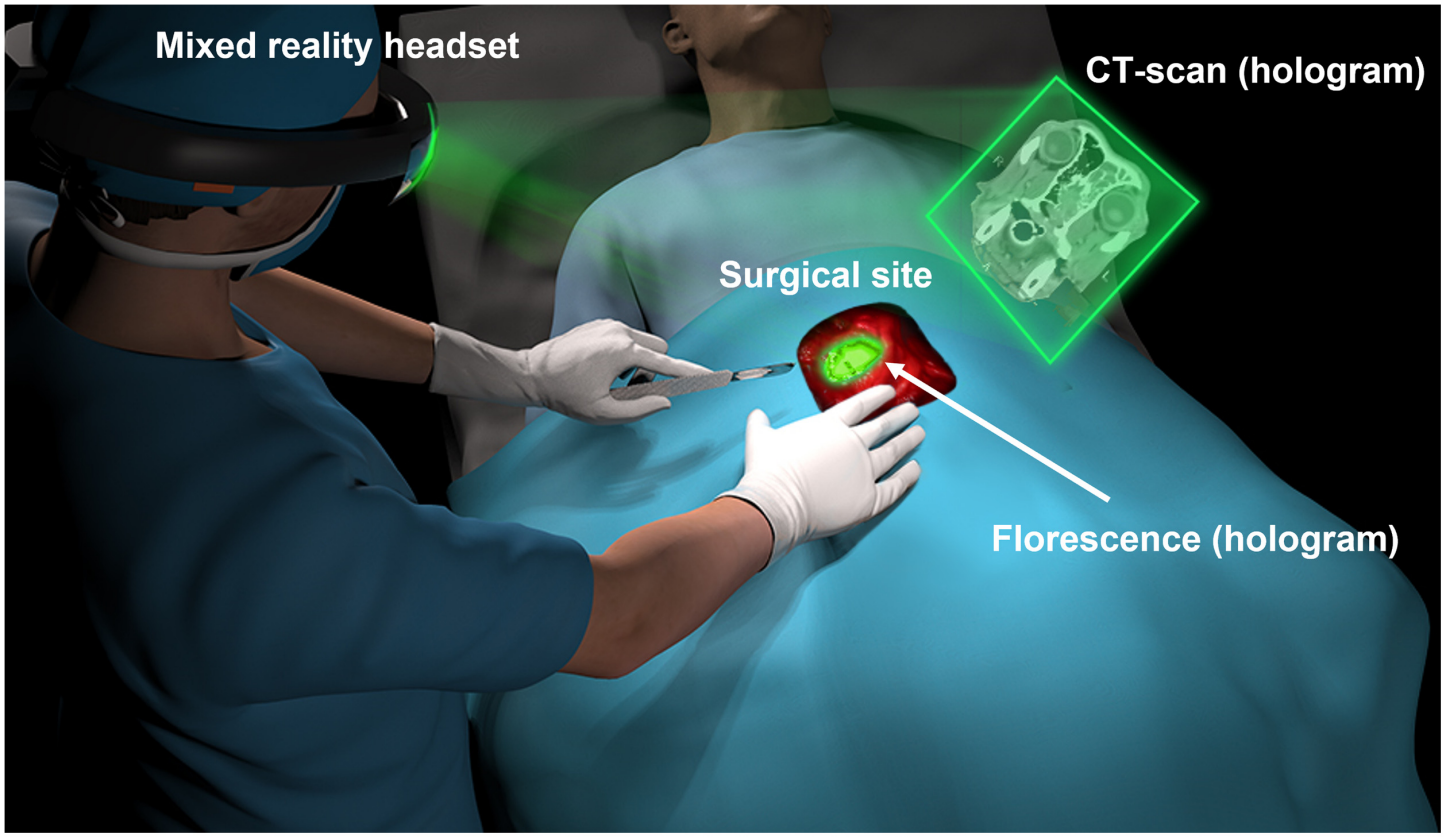
Illustration of our intraoperative imaging system using holographic display, which includes a bioinspired single-chip multispectral imaging sensor, a laser light excitation source, holographic goggles, and a computer for data acquisition and image processing. The system enables visualization of the surgical site with natural eyesight and augmentation of holograms indicating the location of tumor tissue marked by fluorescent molecular probes.

We have developed a complete intraoperative imaging and display system based on holographic goggles to address issues related to real-time fluorescent and depth display information.^31^ The holographic goggles utilize see-through glasses to provide natural vision that is overlayed with computer-generated visual information displayed via spatial light modulators to both eyes, thus creating 3D augmented reality as illustrated in Fig. 1. Incorporated within the holographic goggles is an inertial measurement unit (IMU), which records the user’s head movement and enables the augmented reality to be updated according to the user’s gaze direction. Our system generates augmented visual data about the location of the tumor by capturing NIR information using a bioinspired, single-chip multispectral imaging sensor. This visual information is calibrated and co-registered with the operator’s natural eyesight, allowing for the surgical site to be viewed without obstruction and enhanced with artificial information indicating the location of the tumor highlighted by the fluorescent molecular markers. Our system also displays preoperative images near the surgical site, allowing for easy access with minimal head movement. In the context of this paper, our primary emphasis lies in capturing fluorescence from a single tumor targeted probe and presenting this information to the end user through a 3D hologram. Furthermore, we conducted a thorough evaluation to assess the accuracy of co-registering the 3D hologram from fluorescent phantoms with natural eyesight and compare it with the co-registration accuracy of 2D display monitors. Additionally, we explored the practical application of the bioinspired multispectral color-NIR image sensor within the surgical workflow of a veterinary hospital. Our evaluation involved intraoperative detection of SLNs tagged with ICG in canine patients diagnosed with head and neck cancer who underwent surgical procedures. The fluorescent information is presented as a hologram to the surgeon while conducting SLN mapping and resection. Discussion and concluding remarks are presented at the end of this paper.

## 2 Materials and Methods

### 2.1 Imaging, Illumination, and Holographic Display Overview

Our intraoperative imaging system is comprised of the following components: a bioinspired single-chip multispectral imaging sensor,^19^ holographic goggles (Microsoft HoloLens, 1st generation), laser light illumination (BWF2-780 and BWF2-660, B&W TEK, Plainsboro, New Jersey, United States), personal computer (Lenovo M Series Tiny PC, i7 Intel Pentium, 16 GB RAM), wireless router (R6220, Netgear, San Jose, California, United States), and custom printed circuit board with NIR LEDs for optical calibration and co-registration. For generating 780 nm illumination, we utilized a laser shaping filter (FF01-769/41 Brightline bandpass filter, Semrock) along with a diffusing lens (ACL2520U-DG15, Thorlabs). Similarly, for producing 665 nm illumination, we employed a laser shaping filter (665.85-1 OD6 Ultra Narrow Bandpass Filter, Alluxa) in addition to a diffusing lens (ACL2520U-DG15, Thorlabs).

**Fig. 2:**
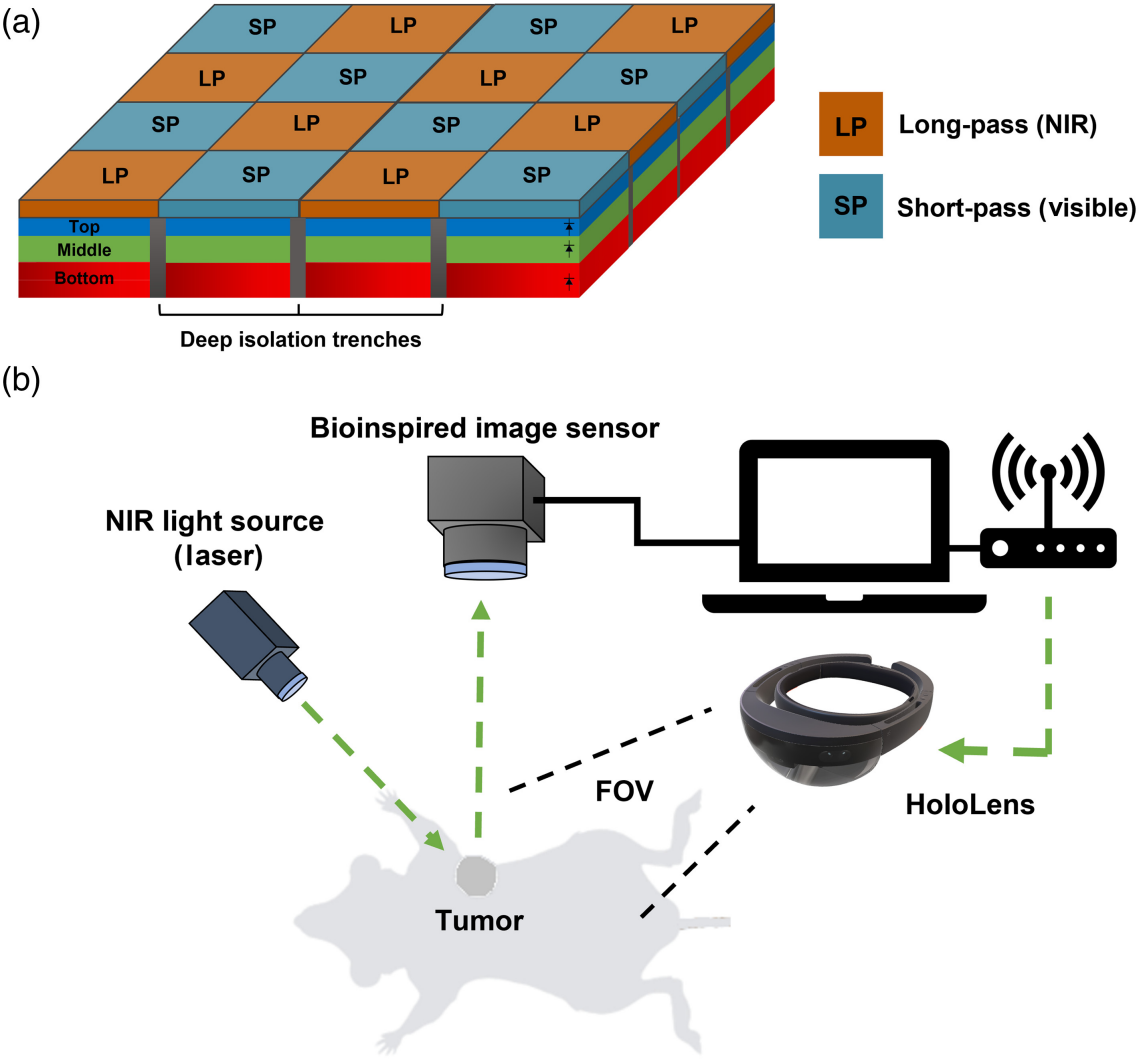
Schematic illustration of the fluorescence-guided surgery system with the bioinspired image sensor and HoloLens. (a) The pixel array of the image sensor with vertically stacked photodiodes, including the top, middle, and bottom layers of photosensitive cells. (b) The experimental setup of the holographic goggles system used for *in vivo* imaging of tumor-bearing mice.

Our bioinspired imaging sensor incorporates a unique design featuring vertically stacked photodiodes combined with pixelated spectral filters [Fig. 2(a)].^19^ The pixelated spectral filter array consists of two distinct pixels arranged in a checkboard pattern across the imaging area. One pixelated spectral filter allows the transmission of photons in the range of 400 to ∼680  nm, whereas the second pixelated spectral filter transmits photons within the range of 700 to 1000 nm. Under each pixelated spectral filter, there are three vertically stacked photodiodes, resulting in a total of six spectral measurements provided by the sensor. Three spectral observations are obtained in the visible spectrum, and the remaining three observations occur in the NIR spectrum. The merged NIR and color image was presented on a standard screen, allowing the surgical staff to view it easily. This facilitated a broader distribution of fluorescent information within the operating room. The multiple spectral measurements in the NIR spectrum allow the sensor to effectively differentiate between multiple fluorescent dyes present in that range. The main focus of this paper is to capture fluorescence specifically from a single tumor targeted probe and deliver this information to the end user through a 3D hologram. Our bioinspired sensor is capable of imaging multiple NIR dyes excited at different wavelengths. This feature was not integrated with the HoloLens and is the subject of future studies.

The bioinspired sensor is housed on a custom printed circuit board with low-noise differential amplifiers, a 14-bit analog-to-digital converter, a low-noise digital-to-analog unit, low-noise voltage regulators, and other auxiliary electronics necessary for the correct operation of the imager. An FPGA board (OpalKelly XEM 7310, Seattle, Washington, United States) is used to read out data from the imager, acquire data from the sensor, and transfer data to the PC. A custom-built camera enclosure houses optical notch filters with optical density >6 to suppress excitation light sources at 665 nm (NF03-658E, Semrock) and 785 nm (NF03-785E, Semrock). All printed circuit boards and the camera housing are designed using Altium or Autocad and fabricated by PCBway (Shenzhen, China). The FPGA firmware is developed using Xilinx Verilog, and the data acquisition and display software are written in the Python and C++ programming languages.

The data from the bioinspired image sensor are streamed to a computer, where additional signal processing is performed; this includes image calibration, co-registration with natural eyesight, color compression, and wireless streaming of data to the holographic goggles to provide augmented information to the surgeon’s natural eyesight about the location of tumors [Fig. 2(b)]. On the holographic display, co-registration with the natural eyesight is performed, and 24-bit color image hologram is generated and displayed. However, high-color resolution images require a long transmission time of more than 250 ms and introduce image lag. Therefore, we explored reducing the bit depth of the color images while still displaying the necessary contrast information to the surgeon under 80 ms from end to end.

A threshold was applied to the acquired NIR image from the bioinspired sensor, and only pixels with digital values above the threshold were presented in false color. Pixels below the threshold were represented as black and hence were rendered transparent on the HoloLens. Hence, the operator views the surgical site with their natural eyesight with only fluorescent information above a certain threshold being augmented to the natural eyesight. The threshold level of the NIR regions can be altered during the surgery to accommodate the operators’ preferences. The compressed NIR image was transmitted wirelessly to the holographic goggles, where an embedded processor generated a false-color hologram using a lookup table and projected it in 3D space. The goggles also have an embedded inertial and magnetic unit to track movements of the user to allow for stabilizing the placement of the hologram position even as the user gaze is away from the surgical site.

### 2.2 Hologram Co-Registration with Natural Vision

Our intraoperative holographic system requires calibration and co-registration of the hologram projected onto the see-through display screen with the natural vision. Initially, the user was directed to launch the calibration application of HoloLens. This step is crucial as it allows for the adjustment of the interpupillary distance to account for the disparity that is unique for each user of the goggle. The application accomplishes this by guiding the user through a series of finger alignment tasks. Before initiating the co-registration procedure, it is necessary to position the camera above the surgical bed in the operating room for imaging. Subsequently, the co-registration process, which consists of two steps, can be performed. First, a custom printed circuit board with four NIR LEDs placed in assigned positions was imaged using our bioinspired sensor. To ensure that all LEDs were captured within the field of view of the camera, it was necessary to select an appropriate working distance (WD). However, once the WD was determined, the calibration routine proceeded regardless of the specific WD selected. A threshold was then applied to the NIR image, and the locations of the four LEDs were determined by analyzing the contours and its first moments. A homography matrix was then computed using the first moments and pixel locations, which were applied to all frames acquired from the image sensor. The assembled hologram was wirelessly transferred to the holographic goggles and displayed to the user’s natural vision.

The second step involves the operator placing the hologram on the calibrated board such that the visual information from the LEDs is correctly superimposed with the hologram. This was achieved using a series of voice and gesture commands that are recognizable by the holographic goggles. This step is a critical process that ensures that the augmented reality provided by our system accurately aligns with the natural eyesight of the operator, enabling them to locate and remove cancerous tissues. Typically, the calibration and co-registration procedure is required to be performed once for continual ongoing usage and takes ∼10 to 15 min to complete. However, if the surgeon removes the wearable goggle, the co-registration process may need to be repeated to account for any changes in the position of the goggles relative to the surgeon’s eyes.

### 2.3 Co-Registration Error Between Holograms and Natural Vision

We conducted an experiment to assess co-registration accuracy between holograms and the natural vision at varying depths. Although the holographic lenses provide a 3D mesh of the real world, it lacks the necessary spatial resolution for intraoperative procedures. We addressed this issue by utilizing planar holograms that were positioned at a fixed distance from the user, resulting in co-registration errors that varied according to changes in distance. We used the calibration printed circuit board and placed the hologram at different offset positions from the calibration board plane. The co-registration error was evaluated as the hologram offset from the calibration board plane, which was incrementally adjusted in 5 mm increments, while the operator’s viewing angle remained fixed at ∼60  deg.

#### 2.4 Hologram Color Rendering for Real-Time Visualization of Fluorescent Targets

Our system was designed with the aim of minimizing the delay in displaying the captured NIR image on the goggles. To achieve this, we evaluated the quality of the virtual image and real-time display on the holographic lenses after different levels of compression. To do this, we generated a 24-bit horizontally modulated gradient image, which was then compressed to 12-bit and 6-bit images (excluding the alpha channel) by extracting the most significant bits. These color-compressed images were transmitted to the holographic goggles for display. The fluorescence image displayed on the holographic display utilizes the alpha-red-green-blue texture format, with the alpha channel controlling the transparency setting. Each channel uses the same bit depth in the final rendering. The fluorescence information, contained within the RGB channels, was selected to be either 4 bits per channel (for 12-bit compression) or 2 bits per channel (for 6-bit compression), resulting in either 16-bit depth or 8-bit depth images, including the alpha channel, respectively.

We utilized mixed reality capture to record the hologram displayed on the goggles and assessed both the quality of the color gradient image after compression and the transmission time between the computer and the goggles for real-time display.

### 2.5 FGS Simulation Study

To evaluate the impact of 2D display monitors and 3D holograms on depth perception while displaying fluorescent information to an end user, we conducted a study using six 3D-printed phantoms with varying levels of hills and valleys. The phantoms were painted black to minimize visual cues to identify the 3D topology. Each valley was filled with 100  μL of ICG, which fluoresced under 780 nm laser excitation ($20\;\mathrm{mW}/{\mathrm{cm}}^{2}$) and was detected by the bioinspired image sensor.

We recruited four participants with a minimum of 5 years of experience in animal handling, including administering tumor-targeted dyes and performing tumor resections. The participants were asked to locate and delineate the borders of the fluorescence signals on each of the phantoms using a pointed sharpie, either by viewing information displayed on a 2D monitor or using our 3D holographic system. A curtain was installed to ensure that the participants only relied on the 2D monitors having monocular depth to make decisions. The distance between the participant’s estimation of the target location and the actual depth was recorded for each phantom.

#### 2.6 Animal Study

##### 2.6.1 Small animal model for breast cancer

We purchased eight female immunodeficient mice (J:NU, 2 months old, average weight 25 g) from the Jackson Laboratory (Bar Harbor, Maine, United States) and inoculated them orthotopically with 4T1 mammary carcinoma cells ($1\times 10^{6}$ cells per injection) into the mammary fat pad. Once the tumors grew to 10 mm in size, each mouse was intravenously injected retro-orbitally with one of the following NIR fluorescent dyes: IRDye 800CW, IRDye 800CW 2-DG, IRDye 800CW RGD, and ICG. After 6 h, each mouse was anesthetized with mask inhalation using 1.5% to 2.0% isoflurane and placed on a stage with a heating pad (37°C). The NIR fluorescent dyes were excited using a 780 nm laser ($20\;\mathrm{mW}/{\mathrm{cm}}^{2}$), and the exposure setting was adjusted based on the fluorescent signal from each dye. The bioinspired imaging sensor was employed to capture NIR fluorescence emitted from the surgical site. The captured data were subsequently compressed and wirelessly streamed to the holographic display, where they were projected as a hologram. To provide a seamless viewing experience, a threshold was applied to selectively project regions with enhanced fluorescence onto the display. This enabled users to perceive the real world with natural eyesight. The specific threshold setting varied between mice depending on the accumulation of dye at the tumor site. The mixed reality capture tool was used to capture visible images superimposed with the NIR-fluorescence images. Although the surgical site was viewed through the holographic goggles’ see-through display, we used the front-mounted color camera to capture visible images for illustration purposes.

After *in vivo* imaging, the mice were euthanized, and the tumors and adjacent muscle tissues were harvested for *ex vivo* imaging using the system. The specimens, which were 10  μm in thickness, underwent staining with hematoxylin and eosin (H&E) and were subsequently imaged using the NanoZoomer slide scanning system (Hamamatsu, Japan). All animal experiments were performed under protocols approved by the University of Illinois Institutional Animal Care and Use Committee (IACUC, Protocol ID 20194).

##### 2.6.2 Intraoperative imaging of canine patient

We evaluated our imaging system in the surgical suite at the University of Illinois Veterinary Teaching Hospital (Urbana, Illinois, United States) using seven canine patients with spontaneously occurring head and neck cancer. Each patient underwent surgical removal of the primary tumor followed by SLN mapping for cancer staging. Before surgery, the holographic goggles were calibrated and co-registered to the operators’ natural eyesight. A 400  μL ICG solution (645  μM) was administered near the tumor site at the start of the surgery, and the site was massaged for ∼10  min. The surgeon then resected the primary tumor, and our intraoperative holographic system was used to determine the location of the SLNs by superimposing fluorescent information recorded by the bioinspired sensor onto the operator’s natural vision. Two other surgical assistants concurrently wore individual holographic goggles, enabling active discussion and training between the primary and resident surgeons. All resected SLN samples were examined on the back table for fluorescent activity and were examined by veterinary pathologists.

## 3 Results

### 3.1 Co-registration of Hologram with Natural Vision

Hologram co-registration with the operator’s natural vision is an important system requirement to ensure accurate localization of fluorescent targets. In the previously published study, we utilized affine transformation to ensure that the Euclidean distances between the LEDs on the calibration board matched the distances between the corresponding regions on the hologram.^31^ However, affine transformation, which preserves line parallelism and ratios of distances along parallel lines, does not account for the effects of perspective distortion. In this work, we were able to address this concern by implementing projective transformation or the homography technique.

**Fig. 3:**
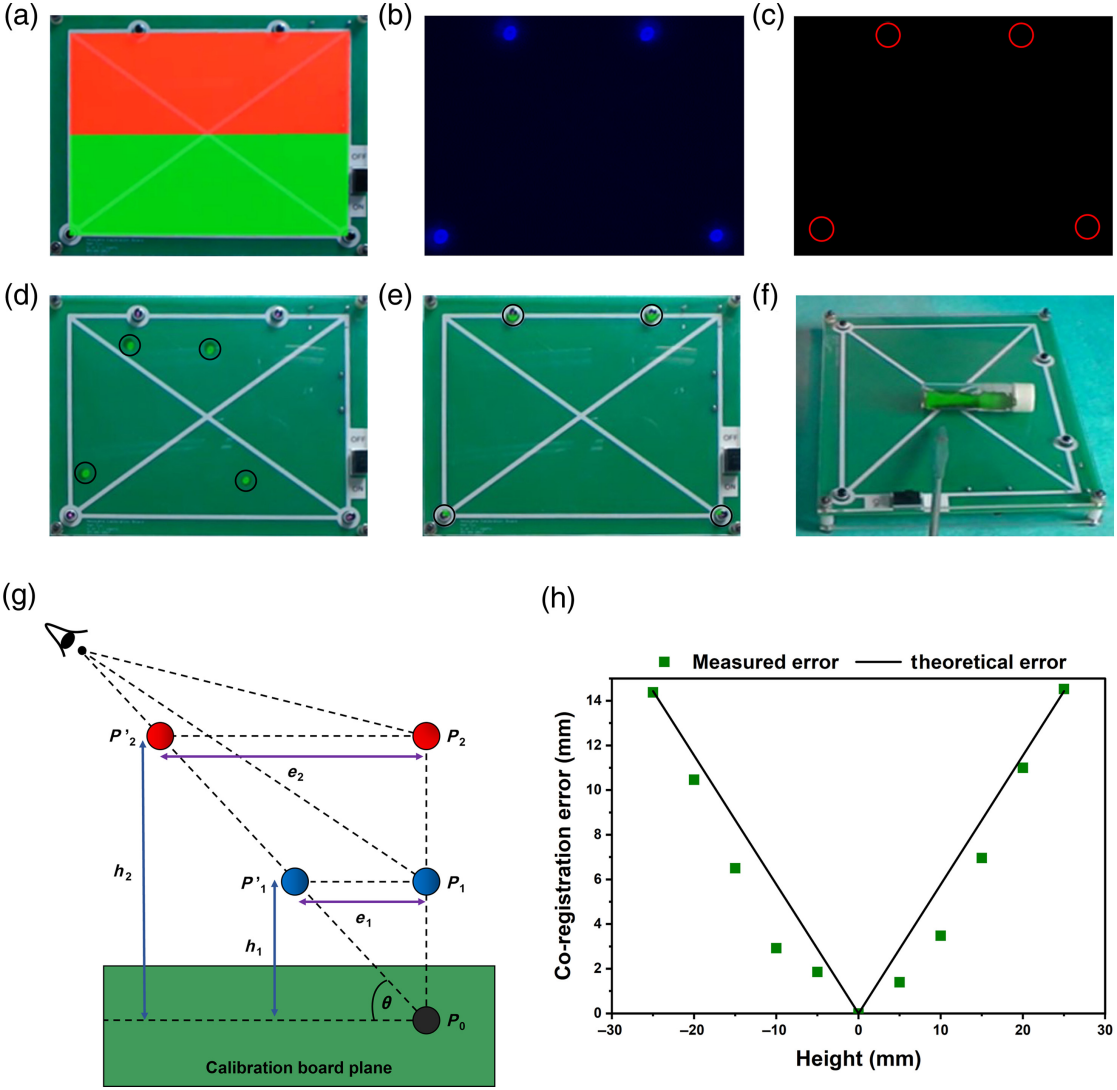
Procedure for co-registering hologram with natural vision, which consists of several stages. (a) The hologram is displayed within the borders of the calibration board. (b) Four NIR LEDs mounted on the board are detected by the bioinspired camera. (c) The detected NIR regions are outlined. (d) Co-registration before calibration and (e) after calibration is illustrated. (f) Co-registration is demonstrated using a vial of quantum dots. (g) The co-registration error model is shown, displaying the dependence of error ($e_{i}$) on the offset from the calibration plane ($h_{i}$) and viewing angle (θ). (h) The error computed from the error model is compared with results obtained from the chessboard pattern at different offsets from the calibration board plane.

Figures 3(a)–3(e) display the various stages of the co-registration routine. First, the hologram was positioned and aligned inside the perimeter of the calibration board [Fig. 3(a)], which consisted of four NIR LEDs located at predetermined relative positions. Next, the NIR signals from the LEDs were captured by the image sensor [Fig. 3(b)]. The contours of each of the detected regions, which were used to compute the homography, are displayed in Fig. 3(c). After calibration, the hologram was co-registered with the locations of the LEDs on the board. Figures 3(d) and 3(e) display the composite image of the calibration board and hologram before and after the co-registration routine, respectively. Figure 3(f) shows the outcome of the co-registration routine using a vial of excited quantum dots. The fluorescent signal displayed in false green was co-registered with the location of the quantum dots within the vial.

We then assessed the co-registration error at different depths relative to the calibration board. Figure 3(g) illustrates an error model describing how co-registration of natural vision with the hologram deteriorates as a function of hologram offset from the calibration board plane and the view angle relative to the same plane. We conducted an experiment to evaluate the co-registration accuracy by recording the distance between the center of a specific LED of the calibration board and the corresponding point in the hologram that was offset from the calibration board plane in increments of 5 mm. The view angle was maintained at ∼60  deg. Figure 3(h) displays the theoretical error along with the measured errors. The equation below describes the mathematical model that quantifies the co-registration error ($e_{i}$) between the real-world point $P_{i}$ and its projection onto the hologram $P_{i^{\prime }}$, as a function of the hologram’s offset from the calibration board plane ($h_{i}$) and the operator’s viewing angle (θ) relative to the plane:

$$e_{i}=\frac{h_{i}}{\tan\;\theta }.$$

Based on this equation, for a given offset, the co-registration error is also influenced by the viewing angle. A viewing angle closer to 90 deg would result in a smaller error, given a specific offset. A good correlation was observed between the model and the observations from the study. The co-registration error linearly increased as a function of the offset of the hologram from the calibration board. When the planar hologram was offset by 5 mm from the calibration board plane, a co-registration error of 2 mm was observed between the center of the LED and its corresponding location in the hologram. In a surgical setting, the co-registration error will vary based on the offset between the region with strong fluorescence and its corresponding area within the hologram. Hence, it is crucial to make precise manual adjustments to the hologram’s position before starting the procedure to minimize this offset and effectively reduce the co-registration error.

### 3.2 Image Compression for Real-Time Display on the HoloLens

**Fig. 4:**
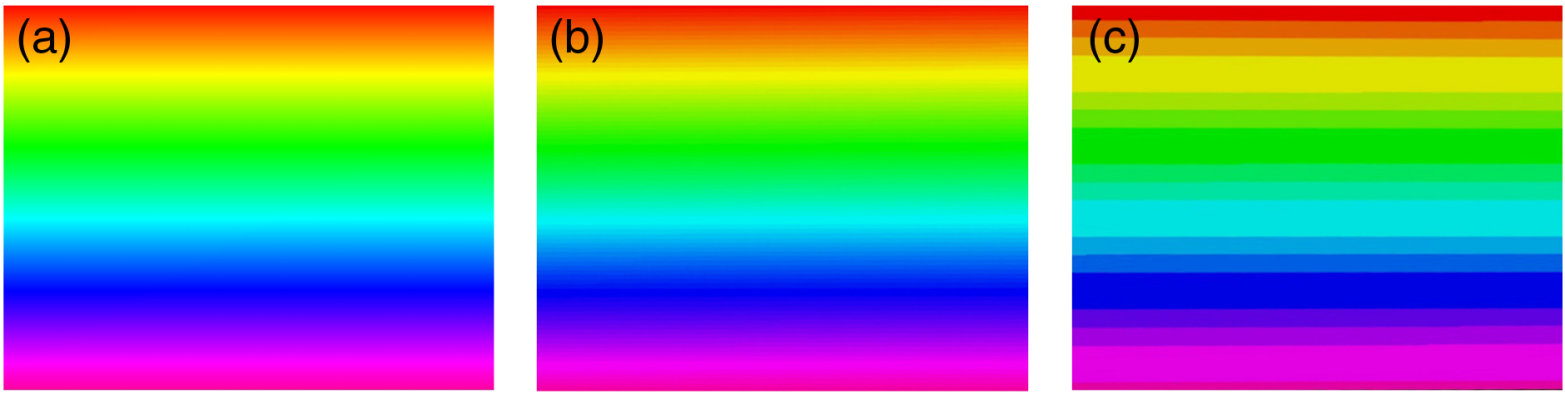
HoloLens displays color gradient images with different bit depths. (a) The original image with 24-bit depth, (b) the image compressed to 12-bit depth, and (c) the image compressed to 6-bit depth. The 12-bit image provides a good balance between reducing data transmission and maintaining a rich and continuous color gamut for the rainbow target.

To reduce the delay between image capture from the bioinspired sensor and image display on the HoloLens, the 24-bit NIR image (excluding the alpha channel) was compressed. We assessed the quality of the image displayed on the HoloLens by visually evaluating it after compressing it to 12- and 6-bit depths. The color gradient image, used for the test, was projected onto the HoloLens before and after the image compression, as shown in Fig. 4. The original 24-bit image displayed a wide color range with seamless transitions between colors [Fig. 4(a)]. The hologram captured from the HoloLens, using the mixed reality capture tool, demonstrated that the system could preserve the image quality when the original 24-bit image was compressed to 12-bit as the color transitions appeared continuous [Fig. 4(b)]. However, when the image was further compressed to 6-bit [Fig. 4(c)], significant degradation in the image quality was observed with discrete transitions between colors. In a surgical setting, it is essential to display a wide color gamut to differentiate regions with high fluorescence from those with low fluorescence. Qualitatively, we demonstrated that we were able to achieve this requirement satisfactorily with 12-bit compression, resulting in an average delay of 50 ms between image capture and display on the HoloLens. Consequently, we adopted the 24-bit to 12-bit compression scheme for all of our studies.

### 3.3 Evaluating Target Delineation Accuracy with Holographic versus 2D Monitor Displays

**Fig. 5:**
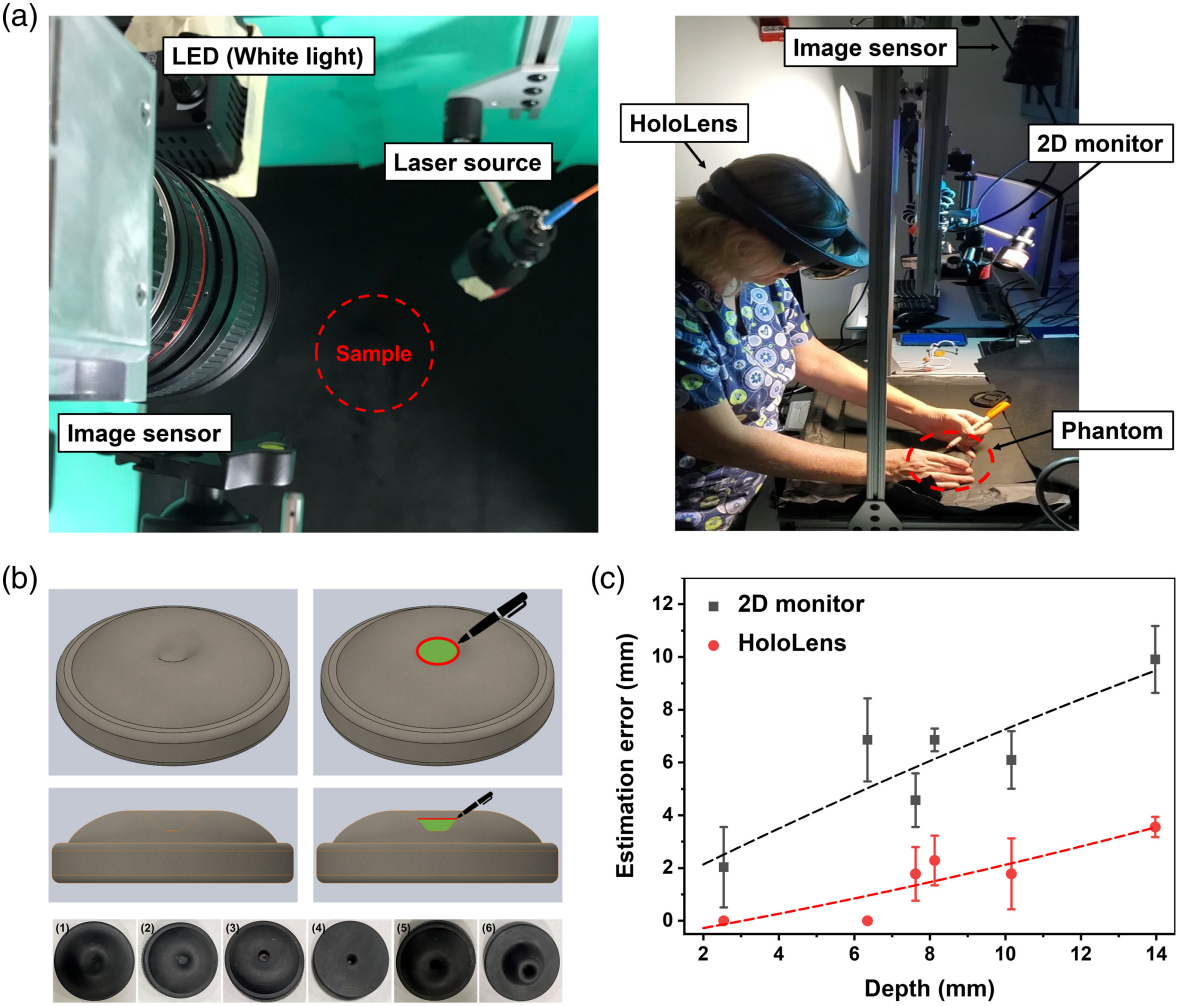
(a) Imaging setup for the simulation study. The left image illustrates the positioning of the camera, illumination optics, and the estimated placement of the phantom (indicated by the red outline labeled as “sample”). The right image showcases a study involving a participant using the holographic display. (b) Phantoms with varying valley depths used in the fluorescence-guided surgery simulation study. The bottom images show the phantoms arranged in increasing order of valley depth. (c) Results comparing estimation error of fluorescent dye location using the 2D monitor and the holographic goggles. The plot shows the mean and standard deviation of the error for each phantom as a function of valley depth, indicating that holographic goggles with stereoscopic vision offers better depth perception compared with 2D monitors.

A longstanding question in fluorescence-guided surgery is whether 3D display goggles provide better localization accuracy of the tumor/target location compared with 2D display monitors and how to measure these differences. Our simulation study involved a set of 3D-printed phantoms that consisted of various hills and valleys to simulate a complex navigational environment. Figure 5(a) illustrates the imaging setup employed for the study with the participant utilizing the holographic display. Figure 5(b) depicts one of the phantoms employed in the study. To improve the visualization of the phantom’s underlying geometry, texture was incorporated into the rendering. The lower images in Fig. 5(b) showcase the actual phantoms utilized, arranged in ascending order of depth. The width and depth of each phantom were randomly chosen. To minimize visual cues about the 3D shape of the object, the phantoms were painted with solid black and the valleys were filled with 100  μL of ICG solution. The phantom was then placed in a simulated operating room installed with the imaging sensor and light source. The task for the participant was to mark the boundary of the fluorescent target using a marker while avoiding markings outside the target area.

We enrolled four participants who possessed a minimum of 5 years of experience in animal handling, which involved tasks such as administering tumor-targeted dyes and conducting tumor resections. Each operator was assigned a phantom randomly. Initially, participants were instructed to perform the task using the information displayed on a 2D screen, where NIR fluorescence images were superimposed on color images. Subsequently, the operators repeated the task using the holographic display, where the NIR fluorescence images were projected as holograms after undergoing the calibration process.

To eliminate any visual biases toward the target that may assist in its delineation, a small curtain was placed between the operator and the phantom when fluorescent information was displayed on the 2D monitor. This experimental setup allowed us to simulate a scenario in which the surgeon solely relied on real-time color and fluorescent information captured by intraoperative sensors when making decisions during surgery. For instance, during lung cancer surgeries involving the resection of small residual tumors marked by tumor-targeted fluorescent probes, the surgeon typically depends exclusively on information provided by intraoperative instruments. However, it is important to note that our experimental setup does not fully replicate scenarios in which the surgeon relies on a variety of cues, including natural vision and intraoperative imaging tools, during SLN mapping or large tumor resection.

Figure 5(c) displays the mean and standard deviation of the estimation error versus the depth of phantom, which were obtained after measuring the distance between the actual location of the ICG solution in the phantom and the participant’s estimation of its location. The plot shows the estimation error for each phantom as a function of the valley depth. The results demonstrate that the participants were able to locate and delineate the fluorescence signal accurately when the valley depth was shallow, resulting in minimal estimation errors. However, an increase in the valley depth led to a proportional increase in the error, indicating impairment of the participant’s depth perception. In contrast, the holographic goggles featuring stereoscopic vision notably reduced the error by providing binocular depth cues that resemble the human visual system in a natural environment.

### 3.4 Imaging Small Animal Models of Breast Cancer with Holographic System

We employed the imaging system to assess *in vivo* imaging and holographic display in small animal models with breast cancer. In a previous study, we conducted an evaluation of the sensitivity and specificity of the single chip, bioinspired imaging sensor for the *in vivo* detection of prostate tumors using single or multiple tumor-targeted probes (IRDye800CW 2-DG and IRDye680RD EGF).^19^ This assessment was performed on seven murine models of prostate cancer. When either one of the tumor-targeted probes was administered to the tumor-bearing mice, the area under the curve was ∼75% due to intratumor biomarker variations. However, when both probes were used together to account for tumor heterogeneity, the area under the curve improved to ∼92%. These experiments confirmed the imaging sensor’s capability to image multiple fluorescent probes with a single imaging chip and improve cancer detection statistics.

**Fig. 6:**
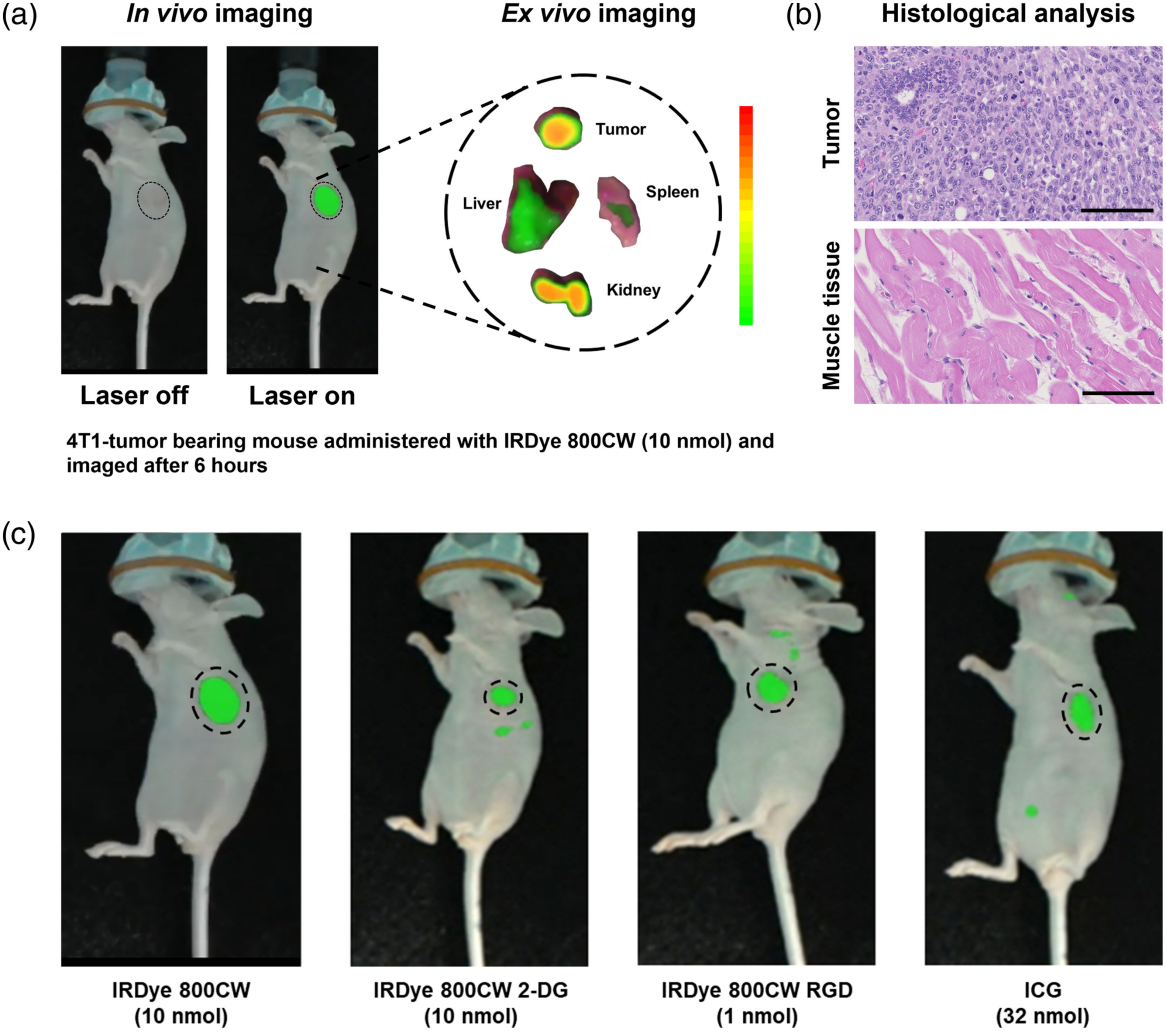
(a) Holographic display of fluorescence image of a tumor-bearing mouse with excitation source turned off and on. (b) *Ex vivo* fluorescence image of resected tumor, liver, kidneys, and muscle tissue with corresponding H&E-stained histological images of a tumor and muscle tissue. Scale bar: 100  μm. (c) *In vivo* fluorescence images of mouse models with breast cancer captured by the HoloLens 6 h post-administration of various NIR fluorophores, including IR800CW (10 nM), IR800CW-2DG (200  μM), IR800CW-RGD (20  μM), and ICG (640  μM), demonstrating the system’s adaptability to various clinical applications (Video 1, MP4, 15.8 MB [URL: https://doi.org/10.1117/1.JBO.28.9.096003.s1]; Video 2, MP4, 6.83 MB [URL: https://doi.org/10.1117/1.JBO.28.9.096003.s2]; Video 3, MP4, 8.97 MB [URL: https://doi.org/10.1117/1.JBO.28.9.096003.s3]; and Video 4, MP4, 11.3 MB [URL: https://doi.org/10.1117/1.JBO.28.9.096003.s4]).

Subsequently, we demonstrated the detection and holographic display of fluorescence, co-registered with the tumor locations, using our system *in vivo*. This was accomplished by utilizing tumor-bearing mice labeled with ICG and three distinct types of IRDye 800 (native, 2DG-conjugated, and RGD-conjugated). The mice were imaged using our holographic system 6 h post-administration of the NIR fluorescent dyes. Figure 6(a) demonstrates the co-registration between the holographic fluorescence image and the tumor location, both with and without the laser excitation sources activated. Following the resection and removal of the tumor, the virtual fluorescence signal, which had been calibrated and superimposed on the tumor, continued to accurately track its location, as shown in Video 1. The fluorescence signal is highlighted in false green to enhance its visibility for demonstration. However, the residual fluorescence within the surgical cavity was not further evaluated in this study as our objective was focused solely on demonstrating co-registration of holograms to tumors during intraoperative settings.

Videos 2–4, featuring two mice, also exhibit the co-registration of the holographic fluorescence signal projected onto the display. Videos 2 and 3 showcase both translation and rotation along the vertical axis (yaw), and Video 4 mainly demonstrates rotation along the horizontal axis (pitch), resulting in variation in the WD. Despite rotational or translational movements, the position of the perceived hologram by the user remained unchanged and correctly superimposed on the tumor tissue. The co-registered hologram consistently aligned with the natural vision, regardless of changes in perspective. It is crucial to note that the observed slow translation and rotation motion can be attributed to the relatively low frame rate of the image sensor, which operates at ∼4 frames per second. This is partly caused by factors such as the low accumulation of fluorescent probes in the tumor, weak laser excitation, and the sensitivity of the camera. Fluorescence emitted by the NIR probes was successfully detected in all eight mice, and this information was accurately superimposed at the appropriate anatomical location in every case. As a result, we achieved a 100% positive predictive value for the detection and display of fluorescent information in the murine model of breast cancer.

The *ex vivo* biodistribution study revealed varying levels of fluorescence in the harvested organs, demonstrating the preferential accumulation of the dyes at the tumor site and their clearance via the liver and kidneys [Fig. 6(b)]. Histological images of H&E-stained specimens of a tumor and healthy muscle tissue indicated the distinct cellular and tissue structures that confirmed malignancy in the specimen identified as tumor. Finally, the *in vivo* fluorescence images in Fig. 6(c) highlighted the system’s ability to detect and display the location of tumors with different types and doses of NIR fluorescent dyes, indicating its potential for various clinical applications. When imaging NIR fluorescence through the skin, as illustrated in Figs. 6(a) and 6(c), the fluorescence signal tends to be weak due to signal attenuation within the skin. As a result, the hologram is depicted in green to represent the lower fluorescent signal. However, in the case of *ex vivo* samples, this issue is not present, and they exhibit a higher fluorescent signal, as indicated by the false color range.

### 3.5 Intraoperative Imaging of Canine Patients

To demonstrate the applicability of holographic display in the operating room, we utilized our holographic goggles to guide surgeons operating on canine patients with spontaneously occurring head and neck cancer. In standard head and neck cancer surgery, an SLN biopsy is performed to determine metastatic progression to the lymph node. The conventional method for the SLN biopsy in head and neck cancer involves the use of fluorophores such as ICG or MB that are administered into the lymphatic system near the tumor site, and surgeons rely solely on their unaided eyes to map and remove the visible fluorophore stained SLNs.^12^ However, it should be noted that both ICG and MB exhibit NIR fluorescence when excited with an appropriate NIR light source. NIR fluorescence has the capability to radiate from SLNs situated just a few millimeters below the surface, rendering this approach more reliable for SLN mapping compared with visible detection.

**Fig. 7:**
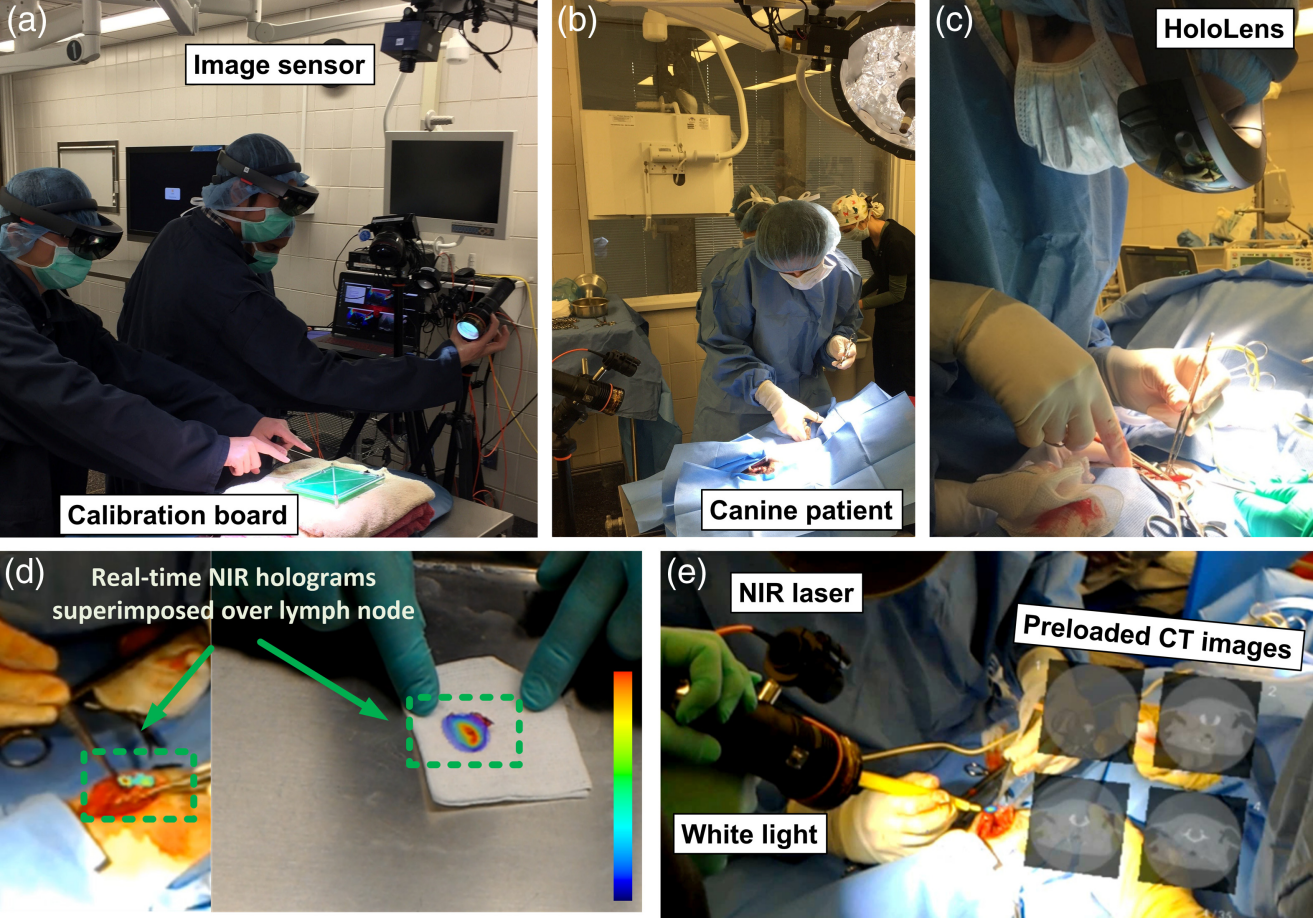
Intraoperative imaging of a canine patient with head and neck cancer using holographic display. (a) Co-registration routine carried out by the clinical staff prior to the surgery. (b) Imaging setup in the operating room with the canine patient at the beginning of the surgery. (c) Veterinarian surgeon wearing HoloLens during the surgical procedure. (d) *In vivo* and *ex vivo* back-table images showing real-time fluorescence holograms (green dotted box) superimposed over SLNs during and after resection, respectively. (e) HoloLens projecting real-time fluorescence and pre-loaded CT images simultaneously as a hologram during the surgical procedure (Video 5, MP4, 28.2 MB [URL: https://doi.org/10.1117/1.JBO.28.9.096003.s5]).

In our study, we employed ICG as the fluorescent dye, which was excited using the NIR light source ∼15  min after its administration. Figure 7(a) demonstrates the co-registration procedure carried out by the clinical staff prior to the surgery. The image sensor was mounted directly above the canine patient as shown in Figs. 7(a) and 7(b). The resulting fluorescence signals were captured and displayed in real time on the holographic goggles to identify SLNs at the surgical site [Fig. 7(c)]. A hologram indicating the location of the SLN was projected and overlaid onto the operator’s visual field. Due to the wireless transfer of images, multiple users, including surgeons and two assistants, were able to access the holographic fluorescence images simultaneously. This facilitated active collaboration and enabled learning during the surgery [Figs. 7(a) and 7(d)]. Back-table imaging of the resected SLNs showed high-intensity fluorescence emitted from the center of the specimen. Both *in vivo* and *ex vivo* composite images demonstrated good co-registration of the NIR fluorescence images with the physical specimen and helped the surgeons to identify and resect the SLNs. After the resection was completed, the holographic lenses were removed from the surgeon by the operating room nurse and the surgical cavity was examined for any additional SLNs. Among the 7 canine patients included in our study, a total of 17 SLNs were detected, out of which 15 demonstrated NIR fluorescence. As a result, we achieved an 88% positive predictive value and 100% negative predictive value for the detection of SLNs using ICG fluorescence and displaying this information on the HoloLens.

Figure 7(e) illustrates projection of pre-operatively acquired CT images onto the holographic display from the perspective of the assistant wearing the holographic display. The image also shows the illumination sources (NIR and white light) used for the surgery. The hologram was able to display both real-time fluorescence images and pre-loaded patient records simultaneously in the surgeon’s field of view during the surgery (Video 5). This showcases the adaptability of our imaging system to meet various surgical requirements in the operating room. The advantages of projecting supplementary pre-operative images include reducing disruptions in the surgical workflow and providing multimodal information that caters to various surgical requirements. Nonetheless, through the use of distinct voice commands, this information can be concealed to prevent any obstruction of the view of the surgical site.

One of the modifications in this setup involves the contrasting viewing perspectives of the surgeon and the multispectral camera, which is positioned above the surgical bedside. This difference in perspective may result in a shadowing effect, potentially leading to inaccurate detection and placement of fluorescent information. Although positioning the multispectral camera near the surgeon’s eyes or making it wearable on their head could help mitigate this issue, it is not feasible due to the camera’s size and the absence of the camera’s IMU information.

## 4 Discussion

FGS has shown great promise in improving surgical outcomes in cancer patients. However, the effectiveness of FGS can be limited by the display system used to visualize the fluorescence signals from tumor-specific probes. This paper presents an approach utilizing holographic display technology combined with a bioinspired multispectral imaging sensor to overcome the limitations of conventional display systems in FGS.

The results obtained from our studies showcased the successful co-registration of the holographic fluorescence signal with both the tumor and SLNs using our system, which combines the bioinspired imaging sensor with the holographic display. These findings were observed in both benchtop experiments and *in vivo* animal studies. The information was presented with minimal interruptions in surgical workflow. The holographic display allows for real-time projection of fluorescence signals onto a see-through display, enabling the surgeon to access the surgical site through natural vision. The system’s ability to display pre-operative imaging data and real-time fluorescence information simultaneously could greatly improve surgical workflow.

We also acknowledge the limitations of our imaging system and the implementation methodology, such as co-registration dependence on view angle and hologram offset from the target due to the use of a planar hologram. Although the current holographic system includes a depth-sensing map that can help address this issue, the resolution of the depth map may not be sufficient for surgical applications. Although the system can be programmed to increase the spatial resolution, this feature is not currently available to developers due to high power consumption, which would limit the battery life of the system. These limitations need to be carefully addressed and optimized before the system can be used in human clinical trials.

## 5 Conclusion

In summary, although progress has been made in improving the sensitivity and specificity of cancer detection using fluorescent probes, the display system in fluorescence-guided surgery has been overlooked. The use of 2D screens leads to loss of depth information, prolongs surgical time, and can result in incomplete resections. This study suggests that combining holographic goggles and bioinspired multispectral imagers can be greatly utilized in the surgical workflow. However, the issue of hologram offset needs to be addressed for wider acceptance of this approach. Further studies are required to fully assess the potential of this technology in clinical settings.
